# Roles of Two Small Leucine-Rich Proteoglycans Decorin and Biglycan in Pregnancy and Pregnancy-Associated Diseases

**DOI:** 10.3390/ijms221910584

**Published:** 2021-09-30

**Authors:** Chidambra D. Halari, Michael Zheng, Peeyush K. Lala

**Affiliations:** 1 Department of Anatomy and Cell Biology, Schulich School of Medicine and Dentistry, The University of Western Ontario, London, ON N6A 5C1, Canada; chalari@uwo.ca (C.D.H.); mzheng69@uwo.ca (M.Z.); 2 Children’s Health Research Institute (CHRI), Schulich School of Medicine and Dentistry, The University of Western Ontario, London, ON N6A 5C1, Canada; 3 Department of Oncology, Schulich School of Medicine and Dentistry, The University of Western Ontario, London, ON N6A 5C1, Canada

**Keywords:** leucine-rich proteoglycans, decorin, biglycan, pregnancy, placenta, decidua, trophoblast, trophoblast invasion, preeclampsia, fetal growth restriction, premature labor, premature preterm rupture of membranes (PPROM), Ehlers–Danlos syndrome (EDS)

## Abstract

Two small leucine-rich proteoglycans (SLRP), decorin and biglycan, play important roles in structural–functional integrity of the placenta and fetal membranes, and their alterations can result in several pregnancy-associated diseases. In this review, we briefly discuss normal placental structure and functions, define and classify SLRPs, and then focus on two SLRPs, decorin (DCN) and biglycan (BGN). We discuss the consequences of deletions/mutations of DCN and BGN. We then summarize DCN and BGN expression in the pregnant uterus, myometrium, decidua, placenta, and fetal membranes. Actions of these SLRPs as ligands are then discussed in the context of multiple binding partners in the extracellular matrix and cell surface (receptors), as well as their alterations in pathological pregnancies, such as preeclampsia, fetal growth restriction, and preterm premature rupture of membranes. Lastly, we raise some unanswered questions as food for thought.

## 1. A Brief Review of the Placental Structure and Functions

Placentas in eutherian mammals have evolved to nourish the embryo and the fetus. For this function, they must invade the pregnant uterus to various degrees.

They can be broadly classified by their degree of invasiveness into the uterine endometrium: (i) “epitheliochorial” type (in ruminants), in which chorionic trophoblast cells remain apposed to the uterine epithelium, without breaching it. There is evidence of a certain degree of fusion between the trophoblast and the uterine epithelium. However, in some regions such as chorionic girdles, trophoblast cells are highly invasive (e.g., in horses); (ii) “endotheliochorial” type (in carnivores), in which the trophoblast invades the uterine connective tissue to reach the maternal endothelium; (iii) “hemochorial” type (in rodents, primates, and humans) in which trophoblast cells invade the maternal arteries to derive oxygen and nutrients from the maternal arterial blood.

### 1.1. Hemochorial Placentation

**Figure 1:**
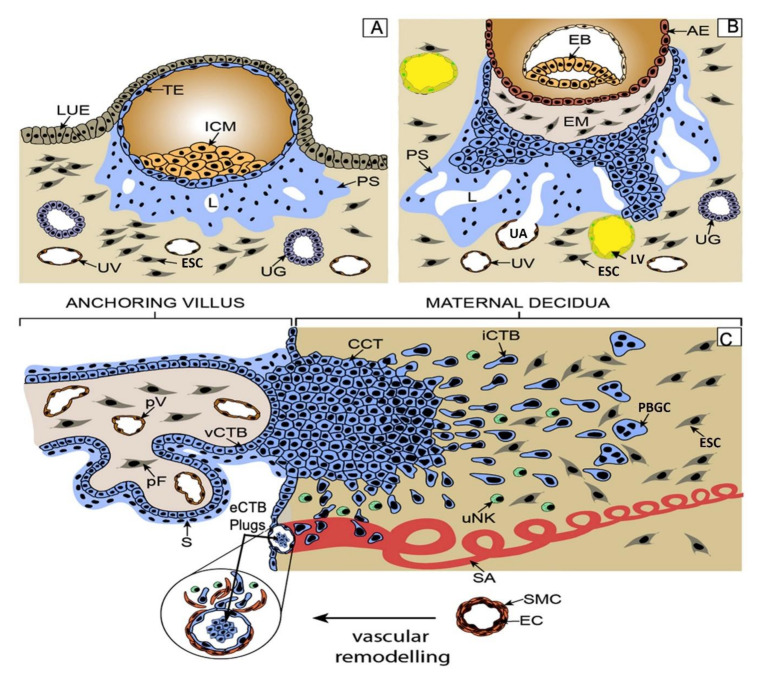
Adapted from [6] with permission. (**A**) Shortly after implantation (end of first week), stem cells of the trophectoderm give rise to the primitive syncytium (PS) by cell fusion. Lacunae (L) develop in the PS, as the precursor of the intervillous space. Invasion of the PS into uterine glands allows “uterine milk” to fill up the lacunae (histotrophic nutrition). Later, with invasion of the PS into uterine vessels (UV/UA), maternal blood fills the lacunae (hemotrophic nutrition). Lymphatics (LL) are also invaded. (**B**) During the end of second week, proliferative cytotrophoblasts (CTBs) break through the PS, forming primary villi. (**C**) During the third week, tertiary villi are formed by migration of the extraembryonic mesoderm followed by vascularization. At distal sites, proliferative cell columns are formed which give rise to different invasive extravillous trophoblast (EVT) subtypes. Interstitial cytotrophoblasts (iCTBs) migrate into the decidual stroma and differentiate into placental bed giant cells (PBGCs). Endovascular trophoblasts migrate into spiral arteries and contribute to uterine natural killer (uNK) cell-initiated remodeling of spiral arteries (SA). Bulmer et al. (2020) [7] recently challenged the long-held view that endovascular trophoblasts (eCTBs) replace the endothelium of spiral arteries during arterial remodeling. By immunostaining a large number of chorionic villus biopsy samples, they found that, while sCTB plugs appear within the arterial lumen, arteries were found with either missing or intact endothelium, but never lined by eCTBs. AE, amniotic epithelium; CCT, cell column trophoblast; ESC, endometrial stromal cell; EB, embryoblast; EM, extraembryonic mesoderm; eCTB, endovascular cytotrophoblast; PBGC, placental bed giant cell; ICM, inner cell mass, iCTB, interstitial cytotrophoblast; LUE, luminal uterine epithelium; L, lacunae; LL lymphatic lumen; pF, placental fibroblast; PS, primitive syncytium; pV, placental vessel; SA, spiral artery; S, syncytium; TE, trophectoderm; UG, uterine gland; uNK, uterine NK cell; UV, uterine vein; UA, uterine artery; vCTB, villous cytotrophoblast.

Hemochorial placentas in different species display many similarities and differences in the trophoblast cell types. Soares et al. (2017) reviewed their nomenclature, functions, and molecular signatures in detail [1]. In the human placenta, maternal blood flow toward the placenta for “hemotrophic” nutrition of the embryo is established at the end of the first trimester. Until then, the nutrition of the embryo is “histotrophic”, derived from secretion products (“uterine milk”) of the uterine glands [2,3]. Shortly following implantation at the early hemotrophic stage, the primitive syncytiotrophoblast invades the maternal vasculature, so that blood sinusoidal spaces appear in the sponge-like syncytium. Subsequently, when chorionic villi are formed, bipotent stem cells contained within the cytotrophoblast layer of the villi differentiate into two distinct pathways: the villous pathway, in which cells proliferate and fuse, giving rise to the villous syncytiotrophoblast layer facing the maternal sinusoids, engaged primarily in exchange and endocrine functions, and the extravillous pathway in which cells break out of the villi as discrete cell columns which proliferate at their base [4,5], migrate, and invade the decidua, endometrial glands, blood vessels, and lymphatic vessels (Figure 1).

### 1.2. Routes of Trophoblast Invasion

For many years, trophoblast invasion was believed to be restricted to uterine decidua and the spiral arteries. This view was based on a variety of approaches including histology of the evacuated early gestational placentas and placental bed biopsies. This explained the connection of the arteries to the intervillous space of the placenta so that maternal oxygenated arterial blood could flow to the placenta. However, this view has been revised in recent years [8,9] evidenced by the findings of the presence of trophoblast cells in other tubular structures, e.g., the uterine glands, veins, and lymphatics at different stages in pregnancy. “Endoglandular invasion”, noted at the time of implantation, likely allows “histotrophic” nutrition of the embryo with glandular secretion products (“uterine milk”) prior to the establishment of “hemotrophic” nutrition. “Endo-venous invasion”, noted at all gestational ages, guarantees the drainage of fluids from the placenta back into the maternal circulation throughout pregnancy. In addition, “endolymphatic invasion” or invasion of trophoblasts into the uterine lymph vessels (identified by lymphatic endothelium-specific markers) has been visualized very early in gestation. This invasion may play a role in regulating placental fluid pressure. At the end of the first trimester, endoarterial (commonly described as endovascular) invasion of trophoblasts into spiral arteries takes place, enabling hemotrophic nutrition. Furthermore, many extravillous trophoblast (EVT) cells remain in the decidual extracellular matrix (ECM) as interstitial trophoblasts, some giving rise to “placental bed giant cells” (PBGCs), which are believed to be noninvasive. PBGCs have two distinct phenotypes [10]: polyploid single cells containing one or more nuclei in a voluminous cytoplasm, and cell aggregates comprising mononuclear trophoblast cells attached to one another by desmosomes, also having gap junctions (Connexin32 and Connexin43). By contrast, gap junctions are absent in the polyploid single giant cells. It remains unclear whether they arise by cell fusion or endomitosis or both. PBGCs are immunoreactive for human placental lactogen (hPL) and human chorionic gonadotrophin alpha chain (α-hCG) (Figure 1).

## 2. Proteoglycans

Proteoglycans (PGs) represent a structurally heterogeneous family of heavily glycosylated proteins that have undergone extensive post-translational modification with sulfated sugar chains. Their protein cores are covalently attached to one or more (up to 100) glycosaminoglycan (GAG) chains. Depending on their molecular size, they are arbitrarily named as “small” or “large”. They are mostly located in the ECM, including the basement membrane. Only one PG (serglycin) has been found to be intracellular, located most abundantly in the granules of mast cells. Another PG family, heparan sulfate proteoglycans, is mostly associated with the cell surface (transmembrane or glycosylphosphatidylinositol-anchored) or pericellular matrix comprising basement membranes (reviewed by Iozzo and Schafer, 2015) [11]. As a major component of the ECM, PGs serve a variety of biological functions. For example, they serve as storage sites for many growth factors, which are shielded from circulating proteases against degradation. They can bind to other PGs or fibrous matrix proteins such as collagen, to serve as a compression buffer against the stress placed on the ECM. They can stabilize ligand–receptor interactions at the cell membrane, creating potentiated ternary signaling complexes that regulate multiple cellular functions such as proliferation, migration, growth factor sensitivity, and adhesion to the ECM. They can also independently activate various signaling cascades, attenuate the signaling of growth factors, or regulate signaling from intracellular compartments. In contrast to classical ligand–receptor signaling, PG-mediated signaling is often characterized by ligand promiscuity and low-affinity binding [11,12,13,14].

### Small Leucine-Rich Proteoglycans

**Figure 2:**
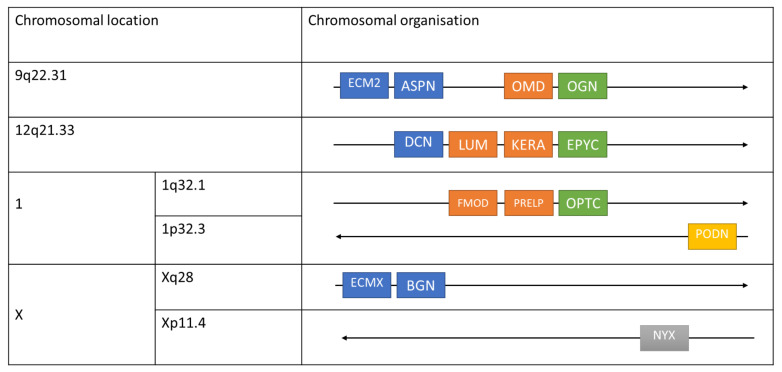
Chromosomal location and organization of various human SLRP classes. The chromosomal arrangement of the various SLRP genes is shown in a telomeric orientation (right). Transcriptional direction is shown by the arrows. The horizontal distance between genes is not to scale. The color-coded dendrogram shows the presence of five distinct families of SLRP. Blue: Class I, orange: Class II, green: Class III, gray: Class IV, and yellow: Class V. This figure was modified and adapted from [11,16].

Small leucine-rich proteoglycans (SLRPs) are a class of PGs characterized by a relatively small protein core (as compared to the larger aggregating PGs) of 36–42 kDa and encompassing a central region constituted by leucine-rich repeats (LRRs). The SLRPs are ubiquitously expressed in most extracellular matrices and are highly expressed during development in the thin membranes enveloping all the major organs such as meninges, pericardium, pleura, periosteum, perichondrium, perimysium, and endomysium. This strategic topology suggests that SLRPs are directly involved in regulating organ size and shape during embryonic development. The 18 SLRP members are grouped into five classes: Classes I–III are coded by canonical genes, whereas Classes IV and V are noncanonical (Table 1 and Figure 2).

SLRPs are ubiquitously expressed in the ECM of most tissues where they act as structural constituents to maintain tissue architecture, and they are involved in range of fundamental biological and pathophysiological functions, including collagen fibrillogenesis [17,18], signal transduction [19,20,21], and tumor-suppressor function [22,23]. SLRPs share many biological functions by binding to ECM components, particularly various collagens, receptor tyrosine kinases, and innate immune receptors (Toll-like receptors) on cell surfaces when present in soluble form [11].

SLRPs are divided into five classes according to several characteristics such as structural and functional properties, homology at both the protein and the genomic level, the presence of characteristic N-terminal Cys-rich clusters, and type of GAG chain [11,21,23,24] (Table 1).

SLRPs are encoded by 18 distinct human genes. In the human, genes encoding SLRPs are spread over seven chromosomes, with some genes clustered on chromosomes such as chromosome 9 or 12 (Figure 2) [21]. At the protein level, SLRPs have a variable number of LRRs comprising the major central domain. The arrangement of these LRRs in the central domain forms a curved, solenoid structure with both convex and concave faces. A typical SLRP consists of an N-terminus, containing four cysteine residues with a class-conserved finite number of intervening amino acids [24,25]. The N-termini in different SLRPS are variably modified to provide unique functions for each SLRP [26,27,28].

Within the conserved C-terminal cysteine-rich capping motif, a distinctive feature is the presence of the recently described “ear repeat”. Typically, the ear repeats consist of 30 or more amino-acid residues including an atypical sequence of Cys located at about 10 residues after the asparagine residue in the consensus LRR [25]. This Cys capping motif, designated LRRCE, is present in Classes I–III (canonical SLRPs), whereas classes IV and V (non-canonical SLRPs) lack this [25]. The ear repeat is proposed to maintain the conformation of the protein core and influence ligand-binding ability, and it is believed to be a hallmark of true SLRP family. A genetic mutation at this position in the *Decorin* (*DCN*) gene (Class I SLRP) leading to a truncated DCN protein core has been found in human congenital stromal corneal dystrophy [29,30]. Most SLRPs have both protein cores and GAG chains. Class I–III SLRPs contain GAG chains with few exceptions, but the noncanonical Class IV and V members lack GAG chains with the exception of 1 SLRP (Chondroadherin). The GAGs of SLRPs are differentially processed in development and aging, and they are variable with regard to size, number, sulfation, and epimerization in different tissues.

Class I—This class contains the SLRP DCN, along with biglycan (BGN), asporin, ECM2, and ECMX. The N termini consist of typical Cys residue clusters that form two disulfide bonds. DCN and BGN contain chondroitin or dermatan sulfate GAG chains. Asporin, although similar to DCN and BGN structurally, lacks the typical Ser–Gly dipeptide and flanking amino acids required for glycanation [16]. Asporin is a member of the SLRP gene cluster present on chromosome 9 with osteoadherin, osteoglycin, and ECM2. ECM2 and ECMX, although structurally different from conventional SLRPs, are 35% identical to the LRR domains of DCN. Similar to asporin, both of them lack GAG chain and are among the two poorly studied Class I SLRPs. ECM2 and ECMX are included in Class I on the basis of genomic and protein homology, although very little is known about their biological functions.

Class II—This class consists of five SLRPs divided into three subgroups on the basis of protein homology. Fibromodulin and Lumican form subgroup A, subgroup B is made of PRELP (proline/arginine-rich end leucine-rich repeat protein) and keratocan, and subgroup C includes osteoadherin. This class contains primarily keratan sulfate and polylactosamine, an unsulfated variant of keratan sulfate. Class II SLRPs have a similar exonic organization (three exons), with the largest exon encoding most of LRRs.

Class III—This class contains three members: epiphycan, opticin, and osteoglycin. In contrast to 10–12 LRRs present in other SLRP classes, the members of this class consist of a low number of LRRs (seven LRRs) and seven exons. Epiphycan is the only dermatan sulfate proteoglycan of this class.

Class IV—This noncanonical class of SLRPs consists of chondroadherin [31], nyctalopin [32,33], and a new member tsukushi [34], which has an expression pattern similar to the shape of the Japanese horsetail plant tsukushi [34]. Nyctalopin was the first described glycosylphosphatidylinositol-anchored member and interestingly was linked to the X chromosome similar to BGN, Class I SLRP. Both tsukushi and nyctalopin have 11 homologous LRRs flanked by an N-terminal Cys-rich region. Tsukushi shares functional properties with class I SLRPs [35,36].

Class V—This is a new, little studied noncanonical class of SLRPs and contains two genes, podocan [37] and a highly homologous podocan-like protein 1 (NCBI accession number 079101). Podocan was originally cloned from a library derived from human immunodeficiency virus transgenic podocytes, giving rise to its eponym [37]. Although these proteins have a different C-terminal Cys-rich cluster, they have 20 LRRs with homology to class I and class II molecules.

Proteoglycan fragments: A number of proteoglycan fragments, including a 32mer IGD peptide of aggrecan, G3 fragments of aggrecan and versican, a G1 containing N-terminal fragment of versican (versikine), C-terminal LG1LG2LG3, LG1LG2, LG3, and endorepellin fragments of perlecan domain V, C-terminal endostatin fragments of type XVIII collagen, a biglycan fragment with transforming growth factor (TGF)-β inhibitory activity, lumican fragments (lumcorin, lumikine), a decorin fragment (decorunt), and a link protein fragment (linkN), have been described. These fragments have cryptic activities ranging from cell proliferative to cell migratory, cell adhesive, cell differentiative, growth factor-like, matrix metalloproteinase (MMP)-inhibitory, tissue repair effects, anabolic matrix stimulatory properties, antiangiogenic properties, and effects on tissue fibrosis. These properties are likely relevant to remodeling of the uterus during pregnancy, but their specific roles in the uterus or the placenta have not been examined. For example, fragments of decorin, biglycan, lumican, and keraotocan have been demonstrated in degenerating cartilages of the human knee and hip joints [38]. As reviewed later, DCN peptides from the LLR-5 domain were shown to be antiangiogenic and migration-inhibitory for trophoblast cells. It remains to be investigated whether they appear in the maternal blood as predictive biomarkers of preeclampsia (PE).

## 3. Distribution and Functions of DCN in the Pregnant Uterus and the Placenta

### 3.1. Cellular Source of DCN and Its Role in Trophoblast Functions

DCN is produced by a variety of stromal cells in the body such as fibroblasts in the dermis, cornea, and chondrocytes of the cartilages [11]. In the pregnant uterus, it is produced by endometrial stromal cells [39], decidual cells, and mesenchymal stromal cells of the chorionic villi [40]. In the latter study, we found that DCN is colocalized with TGFβ in the decidual ECM at various gestational ages. Since we previously showed that decidua-derived TGF-β provides a key control mechanism limiting trophoblast invasion [41], we suggested that DCN in the decidual ECM serves as a storage device for TGF-β in an inactive form, until cleaved and activated by the trophoblast-derived protease cascade at the invasion front, to prevent over-invasion. Surprisingly, however, we found that decidua-derived DCN, on its own, restrained human trophoblast proliferation, migration, and invasiveness, independent of TGF-β [42]. These DCN actions were differentially mediated by binding to multiple tyrosine kinase receptors epidermal growth factor receptor (EGFR), insulin-like growth factor receptor (IGFR)-1, and vascular endothelial growth factor receptor (VEGFR)-2 on the trophoblast cell surface [43]. Further studies revealed that DCN binding to VEGFR-2 is abrogated with a VEGFR-2 blocking antibody, indicating an overlap between the ligand-binding and the DCN-binding domains of VEGFR-2. DCN binding had a 7–10-fold lower affinity than the VEGFR-2-specific ligand VEGF-E or native ligand VEGF-A. Using DCN peptide fragments derived from the LRR 5 domain that blocked DCN–VEGFR-2 interactions revealed that the VEGFR-2 binding site was confined to a 12 amino-acid span of the LLR-5 domain of the DCN protein, overlapping with the VEGF-binding site [44]. VEGFR-2 binding was found to be critical for DCN to restrain VEGF-dependent migration and endovascular differentiation of the trophoblast, an event essential for uterine arterial remodeling during normal pregnancy critical for adequate flow of uterine arterial blood for fetal nourishment [45]. Indeed, mutating a single amino acid at this binding site annulled VEGF-dependent DCN actions [46]. We further discovered that selective DCN overproduction by decidual cells, but not villus mesenchymal cells, was associated with PE with or without fetal growth restriction (FGR), and an elevated level of maternal plasma DCN during the second trimester was a predictive biomarker of PE [47]. We established a cause–effect relationship between decidual DCN overproduction and PE-associated phenotypes: impaired trophoblast invasion, endovascular differentiation, and uterine angiogenesis [48]. Lastly, we discovered that DCN plays a restraining role in human trophoblast stem cell self-renewal and differentiation into syncytiotrophoblast (STB) and EVT [49]. The sources of the DCN are likely both decidual cells and fetal mesenchymal cells.

### 3.2. Localization of DCN in the Uterus and Placenta in the Human and Other Species

DCN is present in both fetal mesenchymal cells and decidua in the human at different gestational ages [40]. While *DCN* is an imprinted gene involved in placental development in mice [50], it is not an imprinted gene for humans [51], pigs [52], and cows [53]. Among cows, *DCN* mRNA expression in the endometrial stroma and chorionic mesenchyme increased during implantation and fell during placentome growth. This drop in *DCN* was reported to impact proliferation, migration, and angiogenesis in the placenta [53]. DCN protein concentration was greater among placentas from parturient cows than first-trimester cows, with higher levels in the fetal portion at both stages [54]. Another time-dependent property of DCN is the composition of the GAG chain. During the latter stages of gestation, a decrease in chondroitin sulfate (CS) and an increase in dermatan sulfate (DS) were noted in the rat placenta [55]. These trends are reminiscent of the shift from a hybrid of CS and DS to DS in aging skin, which coincided with decreased flexibility [56]. In the placenta, an increase in the ratio of type III to type V collagen was noted, which may aid with delivery [57]. Thus, this movement from CS to DS could influence the binding of placental DCN with collagen, inducing flexibility [55].

### 3.3. Role of DCN in Decidualization during Pregnancy

Decidualization is a process that denotes the transformation of endometrial stromal cells (fibroblasts) into specialized polygonal-shaped secretory cells, and it is associated with extensive changes to the extracellular matrix of the uterine stroma. This process has been extensively studied in humans and rodents. In the human, a minor cyclic decidualization occurs during the second half of the menstrual cycle, and it can be induced in rodents artificially after hormone priming [58]. In pregnancy, this process was associated with uterine gland transformation, uterine natural killer (uNK) cell influx, and spiral artery remodeling to maintain adequate blood supply to the growing fetus [59]. The levels of DCN extracted from the uterus was reported to drop during pregnancy until term [60]. The extensive remodeling of uterine ECM that occurs during decidualization has been associated with changes to *DCN* distribution. In mice, decidua begins to develop at the time of blastocyst attachment on gestational day (GD) 4.5 of pregnancy. During the next 3 days of gestation, decidual cells surrounding the site of embryo attachment proliferate and differentiate extensively, eventually becoming larger, often with binucleated or polyploid status [61]. Collagen fibrils with larger diameters up to 250 nm seem to be exclusively present in the decidualized mice uterine stroma [62,63].

An autocrine role of DCN in maturation of spindle-shaped fibroblast-like endometrial stromal cells into large polygonal-shaped decidual cells producing insulin-like growth factor-binding protein (IGFBP-1) and prolactin (PRL)—two well-recognized decidual maturation markers—was shown by Halari et al. (2020) [39]. This was done by knocking out the *DCN* gene in human endometrial stromal cells (HESCs) and exposing them to decidualizing stimuli 8-bromo-cyclic AMP and medroxyprogesterone acetate (MPA). The cells failed to mature as attested by retention of fibroblastic morphology, reduction in polyploidy, and reduced ability to produce IGFBP1 and PRL. Heart and neural crest derivatives-expressed protein 2 (HAND2) and progesterone receptor (PGR) were identified as potential downstream mediators of DCN effects. No role of BGN was detectable as evidenced by the unchanged expression of *BGN* in *DCN*-depleted HESC [39].

### 3.4. Role of DCN in Collagen Fibrillogenesis

The first genetic evidence for the role of DCN in collagen fibrillogenesis was shown in DCN-null mice. These mice displayed abnormal collagen structure in the dermis and the skin fragility phenotype [64]. This phenotype is caused by thinning of the dermis with concurrent reduced tensile strength, a biomechanical impairment directly linked to the abnormal collagen network. Collagen fibrils in decidualized endometrium of DCN knockout (*Dcn^−/−^*) mice displayed larger and more heterogenous diameters, as well as abnormal outlines, compared with wild-type mice [65]. DCN-null mice have tendons with irregular fiber morphology, abnormal fiber diameter distributions, and atypically nonuniform interfibrillar spaces [66,67]. These morphological changes have been attributed to disturbed lateral aggregation of thin fibrils [63]. Thin fibrils, between 10 and 50 nm in diameter, were present in greater quantities among *Dcn^−/−^* mice, suggesting dysregulation or reduced rate of lateral aggregation [65].

### 3.5. Roles of DCN in Matrix Remodeling and Structural Stability of Fetal Membranes

Preterm premature rupture of fetal membranes (PPROM), which is responsible for many preterm births (PTB) [68], has been linked to Ehlers–Danlos syndrome (EDS) [69,70]. A subtype of this syndrome involves a mutation in xylosylprotein-4β-galactosyl-transferase, which results in the secretion of non-glycosylated abnormal DCN [71,72]. The *Dcn^−/−^* mice resemble the cutaneous defects observed in the EDS, characterized by skin hyperextensibility and tissue fragility [73] in a way opposite to fibrosis. EDS patients also experience a heightened risk of aortic rupture [74]. The development of this condition also involves TGF-β signaling [75].

There appears to exist a functional connection between TGFβ and DCN in the murine fetal membranes. At gestational day (GD)12 in mouse, TGFβ, Smad-3, and phosphorylated Smad-3 levels were higher among *Dcn^−/−^* fetal membranes than wild-type. Through a TGFβ-dependent pathway, DCN participates in fetal membrane remodeling during early gestation by upregulating MMPs and downregulating tissue inhibitors of metalloproteinases (TIMPs) [76]. In the absence of DCN, protein expression of MMP-8 and MMP-9 was lowered, while TIMP-1 and TIMP-2 expression was upregulated [76]. At GD18, *Dcn^−/−^* fetal membranes have similar TGFβ levels to wild-type, but levels of Smad-2 and phosphorylated Smad-2 and Smad-3 are reduced. Independent of TGFβ, DCN acts to stabilize fetal membranes by lowering MMP activity during late gestation. Moreover, the abnormal ratio of phosphorylated Smad-2 to Smad in the *Dcn^−/−^* fetal membrane mesenchymal cells was rescued by the addition of a recombinant DCN protein core, but only if the TGFβ receptor was not blocked [76]. The above results show that DCN regulates the balance between fetal membrane matrix remodelling and stabilization during gestation.

Vaginal delivery has been associated with weakened fetal membranes [77], which was not satisfactorily accounted for by decreasing collagen concentration [78]. DCN is the main proteoglycan in the amnion and closely localized with collagen fibrils [79]. A decrease in DCN and collagen was observed in the cervical amnion, indicating that the cervical site is the location of membrane rupture [80]. This finding was consistent with increased quantities of leukocyte elastase and MMP-9, which degrade collagen and DCN, in the cervical membranes [78,81,82]. Considering the upregulation of cyclooxygenase (COX)-2 in term fetal membranes [83], it has been suggested that initiation of labor starts in the choriodecidual membranes [84]. DCN and collagen were plentiful in the fibroblastic portion of the chorion [78], with most DCN mRNA localized in decidual cells for the choriodecidual membranes [84]. *DCN* transcription in the choriodecidual membranes was greater after term vaginal delivery than preterm delivery but lower than before labor. Scarce *DCN* expression after preterm labor suggests that DCN does not simply maintain uterine quiescence [84].

### 3.6. Role of DCN in Uterine Cervix and Myometrium

As a prerequisite for standard vaginal delivery, cervical ripening involves softening, effacement, and dilation. This process involves a slow phase from early gestation to term and a rapid phase around early labor. DCN may promote cervical softening and dilation as observed in term pregnant cervices in which the synthesis and levels of DCN increased [85]. Conversely, a consistent rise in cervical *DCN* expression was noted in rats, with excess DCN disrupting collagen organization and supposedly weakening the tissue [86].

Myometrial *DCN* expression was elevated at term for humans [84] and after spontaneous term and betamethasone-induced premature labor for ewes. This rise in DCN may aid in matrix alterations critical for myometrial activation, with a possible connection between *DCN* expression and myometrial contractibility [87]. Changes in myometrial DCN expression during gestation may suggest an underlying regulatory framework heavily dependent on ovarian hormones. Among nonpregnant ovariectomized ewes, a significant rise in myometrial *DCN* mRNA concentration was only noted after estradiol (E2) treatment, which was not antagonized by progesterone [87]. Furthermore, DCN distribution within the mouse myometrium was primarily influenced by E2 [88].

### 3.7. Role of DCN in Murine Endometrium

In mice, DCN is found in the endometrial stroma even before decidualization. Martin et al. showed that DCN expression was higher in the myometrium during the preimplantation phase and, surprisingly, endometrial localization of DCN was altered with the GD of pregnancy [89]. They observed high DCN expression in the endometrium on GD1 (preimplantation) and DCN loss in the endometrial stroma post implantation on day 5. Since DCN has an antiproliferative property, its loss during the transition of stromal cells to decidual cells might be functionally important [90]. Another study confirmed the presence of DCN in the endometrium even on GD18 of mouse pregnancy [91]. This may indicate that DCN reappears again later in pregnancy, and the change in DCN expression is likely to have a functional significance.

Systemic *DCN*-knockout mice succumb to skin fragility due to disorganized collagen fibers in the dermis layer of skin owing to the need for DCN in collagen assembly [64]. They are fertile but show an upregulation of related proteoglycans including BGN and lumican in the uterus [65]. *DCN*/*BGN* dual knockout mice exhibit reduced litter size, early pregnancy loss [92], and loss of uterine tissue function in a DCN dose-dependent manner [91]. However, these studies did not test whether DCN is critical for decidual development, as shown for maturation of HESC into decidual cells in the human [39].

### 3.8. Role of DCN in Human Endometrium

The human endometrium undergoes extensive remodeling in preparation for implantation [93]. In stromal cell cultures representative of the human proliferative and secretory human endometrium, *DCN* expression was higher in the stromal areas than in the epithelia [94,95]. Deficient levels of DCN were found in the normal proliferative phase endometrium, with a reported drop during the secretory phase and a more remarkable fall during menopause [96]. Upregulation of *DCN* mRNA was observed after proliferative human endometrium cell cultures were incubated with E2 [95]. Likewise, uterine *DCN* mRNA levels rose among ovariectomized mice treated with either E2 or MPA. Only E2 treatment allowed for the deposition of DCN in the superficial stroma of the endometrium [88].

DCN was found to be produced by decidual cells, colocalized with TGFβ in the decidual ECM throughout human gestation [40]. During uterine decidualization, upregulation of human *PRL* and its receptor (*PRLR*) was reported [97,98], alongside *DCN* mRNA. However, PRL limited the rise of *DCN* in decidualized human uterine fibroblast cells, suggesting that PRL acts in an autocrine capacity to inhibit differentiation [98]. In view of the need of DCN for decidual cell maturation, shown earlier [39], these findings suggest a complex regulatory network in decidual development.

## 4. Distribution and Functions of BGN in the Pregnant Uterus and the Placenta

### 4.1. Roles of BGN in Collagen Fibrillogenesis in the Uterus

Mice lacking both DCN and BGN produce abnormal collagen fibrils, characterized by uncontrolled lateral fibril assembly, which in turn results in fibrils with enormous diameters and aberrant profiles [64,99,100,101]. BGN can regulate collagen fibrillogenesis in vivo (cornea, tendon) and in vitro, compensating for the loss of DCN [67,102]. An increased expression of BGN in the non-decidualized endometrium has been observed in DCN-null mice. In these mice, collagen fibrils undergo substantial structural change such as an increase in diameter and the appearance of an irregular profile [65]. BGN displayed similar localization in decidualized tissue for both *Dcn^−/−^* and wild-type mice [65]. Like DCN, it seems that BGN influences collagen fibrillogenesis in the uterine stroma. Thus, in mice, DCN functions can be compensated for by BGN.

### 4.2. Roles of BGN in Matrix Remodeling and Stabilizing the Fetal Membranes

BGN knockout (*Bgn^−/−^*) mice were used to investigate its potential impact on fetal membranes [76]. At GD12, *Bgn^−/−^* fetal membranes displayed similar levels of TGFβ, Smad-2 and 3, and their phosphorylated counterparts, as noted in the wild-type mouse. BGN promotes MMP activity in fetal membrane remodeling independent of TGFβ during early gestation. At GD18, TGFβ and Smad-2 levels decreased among *Bgn^−/−^* fetal membranes compared with wild-type. Through a TGFβ-dependent pathway, BGN works to upregulate TIMPs and collagen α1VI for fetal membrane stabilization in late gestation [5]. Like DCN, the regulation exerted by BGN over fetal membrane matrix is biphasic. In the *Bgn^−/−^/Dcn^−/−^* double-null mice, the expression of TIMP-3, TIMP-4, and collagen α1VI was reduced. While these two SLRPs have complementary roles during gestation, they have distinct effects on TIMPs, MMPs, and collagen α1VI [76].

Changes in BGN may be involved in the modified biomechanical properties of the human fetal membranes. While not the main proteoglycan in term amnions, BGN was mainly located around and within the amniotic epithelium [79]. In the pre-labor amnion, the levels of BGN in the mid-zone region were greater than the cervical region. However, BGN concentration doubled after delivery in the cervical amnion, further supporting it as the site of membrane rupture [80]. BGN was the predominant proteoglycan in the trophoblastic portion of the chorions and the midzone region of the pre-labor chorio-decidua. A significant increase in the BGN concentration within the mid-zone chorio-decidua was noted after delivery, which could account for the heightened extensibility [77]. These findings strongly suggest that BGN contributes to preparing the fetal membranes for labor [79,80].

### 4.3. Role of BGN in the Uterine Cervix, Myometrium, and Endometrium

Functional roles of BGN present in the uterine cervix and myometrium remain largely unexplored. In the human uterine cervix, inconsistent findings on *BGN* synthesis and levels have been reported. While one study documented a rise in postpartum cervices [85], an in vitro study reported a drop in BGN levels in fibroblasts from preterm and term partum cervix [103]. These disparities may be accounted for by the nature of degradation and the number of side chains in BGN core protein [103]. In the mouse myometrium, BGN was not expressed in the absence of ovarian hormones. However, BGN was detected in both the external and internal muscle layers and the connective tissue between the layers after treatment with E2 or MPA [88].

DCN was the dominating proteoglycan in the human uterus, and a smaller amount of BGN was found. A considerable amount of heparan sulfate proteoglycans were also detected. DCN and BGN decreased by 40% until term. The amount of heparan sulfate proteoglycans increased by 46% during active labor. These data indicate that a considerable remodeling of the uterine connective tissue occurs during pregnancy and labor. It is speculated that these changes in the proteoglycans may be important for normal myometrial contractions during labor [60].

Prior to decidualization, BGN was mainly absent from the mouse endometrial stroma but was widely present in the decidual and non-decidualized regions after decidualization [89,104]. The increase in BGN expression may be required to form the appropriate decidual collagen organization [89]. *BGN* was downregulated in the secretory phase [96,105], with a more remarkable fall in menopause endometrial tissue [96]. In support of the role of ovarian hormones, *BGN* expression in ovariectomized mice rose after administration with MPA but not E2 alone. It was also suggested that the deposition of the BGN protein was dependent on E2 [88]. The nonpregnant premenopausal uterus is subject to cyclic structural changes. Using immunohistochemistry, Lucariello et al. (2015) [96] showed that the distribution patterns of SLRPs were completely modified in the pathological compared to normal endometrium. The expression of SLRPs was low/absent in all endometrial pathologies examined compared to normal endometrium. There was an increase in lumican from the proliferative to secretory phase of the endometrium and a decrease in fibromodulin, BGN, and DCN. In menopause endometrial tissue, the level of expression of fibromodulin, BGN, DCN, and lumican dramatically decreased. The results revealed the prominence and importance of proteoglycans in the tissue architecture and extracellular matrix organization.

### 4.4. Role of BGN in Endometrial Decidualization

Studies by San Martin et al. [106], using immunocytochemical electron microscopy, showed that BGN is associated with the presence of thick collagen fibrils in decidualized regions of the endometrium, and that DCN is associated exclusively with thin collagen fibrils in non-decidualized endometrial areas. These results strongly indicate that BGN plays a role in collagen fibrillogenesis and probably participates in the determination of collagen fibril thickness in the mouse decidua. No functional study of BGN in human or murine decidual tissue has been reported to our knowledge (Table 2).

## 5. Mode of Action of DCN

DCN binds to many constituents of the ECM, growth factors, cell surface tyrosine kinase receptors, and Toll-like receptors, leading to a multiplicity of DCN actions.

(a) Collagen: Interaction between the DCN core protein and collagen is needed for collagen fibrillogenesis [107,108], increased fibril diameter [107], and increased tensile strength of fibers [109,110]. As mentioned earlier, the presence of DCN was associated with altered collagen organization in the cervix [85,86], uterus [65,89], fetal membranes [76,79,80], and placenta [55].

(b) DCN interaction with thrombospondin-1 (TSP-1) [111,112] and fibronectin (FN) [113,114] may impede migration of endothelial cells and contribute its antiangiogenic action. DCN-mediated inhibition of VEGF-mediated angiogenesis results from DCN binding to VEGFR2 [45].

(c) Growth factors: The core protein of DCN interacts with TGFβ, with two identified binding sites of different affinities [115]. While DCN was linked to TGFβ inactivation for reduced cell growth [116], it has been alternatively proposed that TGFβ sequesters DCN in the ECM [117]. DCN was also associated with limiting TGFβ-dependent fibrosis [118,119]. We demonstrated that TGFβ was detected in the cytoplasm of the villous syncytiotrophoblast throughout gestation. In the decidual tissue, TGFβ distribution switched from mainly being present in the ECM during the first trimester to intracellular in the decidual cells at term. Given the colocalization of DCN with TGFβ in the first-trimester decidua, we proposed that DCN stores TGFβ to inhibit its antiproliferative and anti-invasive actions. Furthermore, matrix-degrading enzymes secreted from invasive trophoblasts could break up this complex to activate TGFβ [40]. The finding that TGFβ bound by DCN is released after cleavage mediated by MMP-2, -3, and -7 [120] supported our proposed mechanism for controlling EVT invasion. However, we discovered that DCN diminished proliferation, inhibition, and migration of EVT cells independently of TGFβ in vitro and in situ [42,43]. The antiproliferative effect of DCN was associated with p21 upregulation. We also determined that malignant trophoblast (choriocarcinoma) cells resisted the negative regulation of proliferation, migration, and inhibition exerted by decidua-derived TGFβ or DCN [42]. The underlying mechanisms remain to be explored.

(d) Tyrosine kinase receptors (TKR): DCN can bind as an antagonistic ligand to multiple TKRs to exert negative regulatory functions such as proliferation, migration, and invasiveness of human EVT cells [43,48]. As a ligand for EGFR [121,122], DCN prolongs p21 production [123] and promotes caveolar-mediated endocytosis of EGFR [124] for reduced proliferation [125] of cancer cells. While DCN binding to IGF-IR resulted in greater phosphorylation, it also interacted with IGF-1 [126]. The DCN core protein also induced transient phosphorylation and degradation of the MET receptor [127], which downregulated hypoxia-inducible factor 1 α (HIF-1α) and β-catenin. While this interaction reduced transcription of *VEGFA*, *MMP2*, and *MMP9*, it increased *TSP-1* and *TIMP3* for an overall antiangiogenic effect [128]. Furthermore, DCN can inhibit VEGF-induced angiogenesis [129]. Our lab uncovered the binding of the DCN core protein to VEGFR-2, overlapping with its ligand-binding site for VEGF-E and VEGF-A with a lower affinity than its normal ligands. This low-affinity interaction inhibited VEGF-E-induced migration of EVT cells by blocking extracellular signal-regulated kinase (ERK) 1/2 activation [44]. Later work revealed that DCN inhibited VEGF-mediated endovascular differentiation and migration in EVT cells by interfering with p38 mitogen-activated protein kinase (MAPK) and ERK1/2 pathways in parallel [45].

(e) Toll like receptors (TLR): As a ligand for the Toll-like receptors 2 and 4 (TLR2/4) in mouse macrophages, DCN increased the expression of proinflammatory tumor necrosis factor α (TNFα), interleukin (IL)-12, and anti-inflammatory IL-10 through MAPK and nuclear factor-κB (NF-κB) pathways. By binding to and inactivating TGFβ, DCN could also curtail IL-10 abundance [130]. Substantial thrombin inhibition involving heparin cofactor II (HC II) was reported in pregnant women. This elevated anticoagulant activity was attributed to a DS proteoglycan [131], later identified as DCN in the human term placenta [132]. Both of these interactions may account for proposed roles of DCN in immune signaling [133,134] and blocking thrombosis [135] involving several abnormal pregnancies.

## 6. Mode of Action of BGN

Like DCN, BGN binds to constituents of ECM, growth factors, and TLRs but not TKRs.

(a) Collagen: Like DCN, BGN interactions with collagen are proposed to modulate ECM organization. While a lower amount of collagen was incorporated into fibrils in the presence of BGN, no changes in fibril diameter were associated with BGN treatment [107]. These interactions with collagen have widespread implications for cervix ripening [85], decidualization [65,89], and fetal membrane rupture [80].

(b) Growth factors: TGFβ binds to the core protein of BGN with similar affinity and binding sites as DCN [115]. This interaction may impact the TGFβ–bone morphogenetic protein (BMP)–Smad pathway and its crosstalk with the pathway involving Wnt and Wnt-1-induced secreted protein (WISP) [92,136]. The proposed anticoagulant activity of BGN [131] was reaffirmed by BGN promoting thrombin inhibition by HC II [137]. Dysregulation in the above pathways may account for proposed impact of BGN in abnormal pregnancies.

(c) TLRs: As an endogenous ligand of TLR2/4 in macrophages, BGN plays a multifaceted role in inflammation. BGN binding to TLR2/4 activated p38, ERK, and NF-κB signaling, which resulted in a rise in TNFα and macrophage inflammatory protein-2 (MIP-2) levels [138]. TLR2/4-BGN binding was also associated with a rise in C–X–C motif chemokine ligand (CXCL)-1, CXCL2, CXCL13, C–C motif chemokine ligand (CCL)-2, and CCL5 [139,140,141]. Intriguingly, the interaction between TLR2/4 and BGN also upregulated *HIF-1α* and VEGF in endothelial cells [142]. BGN binds to TLR2/4 and purinergic P2X4/P2X7, which activated the nucleotide-binding oligomerization-like receptor, pyrin domain-containing 3 (NLRP3) inflammasome and enhanced the release of mature IL-1β [143]. Through complex interactions involving TLR2/4 and NADPH oxidases (NOX), BGN influenced the synthesis and maturation of IL-1β [144]. The proinflammatory role of BGN deserves investigation, when considering the impact of inflammation in pregnancy [133,145].

(d) TKRs: Unlike DCN, many BGN functions do not appear to be mediated through interactions with TKRs. For example, unlike DCN, BGN did not affect proliferation and expression of p21 through EGFR signaling [123]. However, the interaction between BGN and TGFα was reported to disrupt downstream EGFR signaling, which hindered the migration of mesenchymal cells during eyelid morphogenesis [146]. While the interaction between VEGFA and BGN did not directly activate VEGFR signaling, the reportedly proangiogenic BGN may result from the matrix storage of VEGF [147].

## 7. Role of DCN in Pregnancy-Associated Disorders

### 7.1. Preeclampsia

PE is considered to be the maternal consequence of impaired remodeling of uterine arteries, which is highly dependent on the action of EVT cells. While some investigators reported that DCN was upregulated among PE placentas [148,149], others have concluded that no such difference exists [150]. This disparity may be explained by the extent of decidual tissue included in placental samples. Our lab demonstrated that DCN overexpression in PE was selectively noted in the decidual and not in the chorionic villus mesenchymal cells [47].

PE can be further classified by onset, with early-onset (EOS) PE before the 34th gestational week and late-onset (LOS) PE at the 34th gestational week or later [151]. DCN placental levels were substantially greater in EOS-PE patients than those with LOS-PE [148]. It has been noted that serum DCN levels were higher among PE women during the third trimester [150].

Our lab determined that PE patients had higher plasma DCN levels, including free and fibrinogen-bound DCN, during the second trimester. This alteration existed before the clinical onset of PE, pointing to its promise as a predictive biomarker. Since maternal body mass index (BMI) and body weight do not impact the size of the placenta, the inverse correlation between DCN and these markers does support the decidua as the primary source of plasma decorin during pregnancy [47]. More specifically, DCN mRNA and protein levels were greater among basal plate decidual cells from PE placentas at all stages, with these differences not observed in the villus mesenchymal cells or chorionic villi. These results demonstrate that high DCN levels at the fetal–maternal interface contribute to PE development and reaffirm the maternal origin of this disease [47].

Knocking down DCN in HESCs led to the incomplete restoration of EVT cell invasiveness of stromal cells. This established a causal link between high DCN levels and the hypoinvasive EVT phenotype [47]. These findings are consistent with earlier work showing that overexpression of DCN reduced EVT migratory and invasive capacity along with a decrease in MMP-2 and MMP-9 protein levels [149]. This negative regulation of trophoblast migration and invasion by DCN contributes to the insufficient conversion of maternal spiral arteries [149]. Trophoblast cells overexpressing DCN also experienced decreased proliferation, with cell-cycle arrest occurring at the G1–G0 phase and enhanced apoptosis [149].

### 7.2. Fetal Growth Restriction

FGR occurs when the fetus does not accomplish its in utero growth potential and is often assigned when the birth weight falls at or below the 10th percentile for the fetus’s gestational age and gender. As the fetal consequence of incomplete remodeling of the uterine arteries, one might expect the SLRP trends from PE to also apply to FGR patients. Idiopathic FGR patients were reported to have higher maternal DCN serum levels, which were negatively correlated with birth weight [152]. In contradiction, lowered DCN mRNA and protein expression was detected for idiopathic FGR placentae [153] and primary placental microvascular endothelial cells (PLECs) from FGR pregnancies [135]. Small for gestational age (SGA), which refers to birth weight below the 10th percentile, is often used as a surrogate for FGR [154]. The above placental trend was further supported by lower expression of DCN mRNA and protein among first-trimester SGA placentae [155]. Evidently, further work is needed to resolve the discrepancy in these reports. In our studies, we found that a subset of FGR was associated with high DCN expression in the decidua only in the presence of PE [47].

In the placenta, DCN protein was localized to the microvascular endothelial cells of villous stroma around fetal capillaries [153]. When DCN mRNA and protein expression was reduced in human microvascular endothelial cells (HMVEC) by SiRNA treatment, the cells exhibited impaired vascular network formation in vitro and displayed increased endogenous thrombin levels and decreased proliferation without a rise in apoptosis [135]. These results were corroborated with PLECs from FGR pregnancies [135]. These findings contradict the well-recognized antiangiogenic function of DCN [21,44,45,48]. It remains a mystery whether these results were specific for the particular endothelial cell class used or are VEGF-independent. VEGF is the major angiogenic factor acting by binding to VEGFR-2, and DCN was shown to act as a negative regulatory ligand by binding to VEGFR-2 [44,45].

In PE patients, DCN core proteins were upregulated in the umbilical arteries (UA) [156], which may be related to increased collagen [157], and umbilical veins (UV) of PE patients [158]. These changes in DCN expression may impact the mechanical properties of umbilical vessels along with fetal blood circulation [156,158]. While the expression of collagen types I and III was higher in UAs from FGR pregnancies, differences in *DCN* mRNA levels were not uncovered [159]. Despite the absence of disparities in UA and UV DCN levels associated with idiopathic FGR, meaningful correlations between these quantities were established with UA systolic/diastolic ratio, UA refractive index, and median uterine artery refractive index [152].

### 7.3. Preterm Birth, Preterm Labor, and PPROM

DCN protein concentrations in the fetal membranes dropped at term, with a further fall after labor (both preterm and term) within the amnion. This decline in DCN during the third trimester and labor may aid in mechanical weakening [160]. Consistent with this finding, preterm labor patients displayed lower serum DCN levels. While serum DCN levels alone could predict PTB before 37th gestational week among preterm labor patients, a combined measurement with cervical length was required to predict PTB within 7 days and before the 34th gestational week [161]. PPROM was associated with lower DCN serum levels appearing in the second trimester [145] and a decline in p-Smad-2 [134]. As a possible cause of PPROM [68,162,163], chorioamnionitis was linked to substantial DCN degradation in the amnion [160]. Only in the presence of infection was lower fetal membrane DCN protein expression noted for PTB with PPROM than without PPROM [134]. Inflammation-induced degradation or downregulation of DCN may account for weakened fetal membranes in PPROM pregnancies [134,160].

### 7.4. Endometrium-Related Disorders

DCN has been associated with regulating endometrial organization where its expression decreases from proliferative to secretory phase and is also reduced in endometrial pathologies such as hyperplastic and postmenopausal polyp [96]. Endometrial hyperplasia is common among polycystic ovary syndrome (PCOS) patients, which display greater DCN protein expression in their proliferative phase of endometrial tissue. This phenomenon has been accounted for by excessive estrogenic activity [164]. Endometriosis, which refers to the presence of endometrium-like tissue outside the uterine cavity, can be treated with dienogest, an effective progestin that reduces endometriotic lesions [165,166,167]. Administration of this drug or progesterone in human endometrial epithelial and stromal cells increased *DCN* mRNA expression by promoting progesterone binding to the *DCN* promoters, verified with dienogest-treated endometriosis patients [168].

### 7.5. Invasive Placentas

An abnormally invasive placenta is a property shared by placenta accrete, percreta, invasive moles, and choriocarcinomas. DCN was detected in EVT cells for the above pathologies suggesting that this DCN is a marker for hyperinvasive placentas [169]. Since DCN is a product of mesenchymal cells, this unexpected finding may suggest that trophoblast cells in these hyperinvasive placentas may have undergone epithelial–mesenchymal transition (EMT), a possibility that needs confirmation. Another possibility suggested by the authors is that DCN is a pro-invasive molecule, which has been refuted by many studies showing its anti-invasive function in trophoblast cells [41,149], upregulation in PE-associated decidua [47], and tumor-suppressive function when upregulated in tumor-stroma [170]. Al-Khan et al. (2020) [171] examined hyperglycosylated hCG (h-hCG), decorin, and IL-8 in the maternal plasma from five groups, comprising (1) normal term controls, (2) placenta previa controls, and cases of (3) placenta increta/percreta without placenta previa, (4) placenta previa increta/percreta, and (5) placenta previa accrete; they found no significant differences of these markers in the various PAS groups from the control groups.

## 8. Roles of BGN in Pregnancy-Associated Disorders

### 8.1. FGR

The placentae of third-trimester idiopathic FGR pregnancies were associated with lower *BGN* mRNA and protein expression [172], reconfirmed in first-trimester SGA placental tissues [173]. It has been proposed that lowered placental *BGN* levels may contribute to FGR by promoting thrombosis or structural alterations [172]. Within the placenta, BGN protein expression was localized to endothelial cells and subendothelial cells of the perivascular region of fetal capillaries [172]. Interestingly, reduced BGN expression in telomerase-immortalized microvascular endothelial cells did not meaningfully affect apoptosis, proliferation, or thrombin generation [173]. However, these conditions negatively regulate angiogenic activities, suggesting that BGN is proangiogenic. Furthermore, substantial shifts in mRNA expression were noted in genes related to angiogenesis, with some of these trends reaffirmed in SGA first trimester placental villi [173].

### 8.2. PPROM, PTL, and Endometrial-Related Pathologies

Patients diagnosed with PPROM displayed heightened BGN serum levels during the second trimester, which may represent an immune response to early tissue damage in the fetal membranes [145]. Chorioamnionitis was associated with substantial degradation of BGN, which may account for diminished strength of preterm fetal membranes [160]. While BGN concentration was lower in the preterm amnion, BGN expression was primarily unchanged between the preterm and term chorio-decidua [160]. Both term and preterm labor were associated with increased BGN concentrations in the amnion, which was also connected to mechanical weakening [160]. In endometrial pathologies, BGN expression was lowered in hyperplasias and postmenopausal polyps [96], but a rise was noted in PCOS endometrium [164]. Like DCN, it has been suggested that BGN regulates tissue organization [96].

## 9. Compensatory and Functional Overlaps of DCN and BGN

The uterus remains dormant during gestation to prevent PTB and undergoes active contraction for the expulsion of the fetus [174], with EDS women at increased risk of uterine rupture [175,176]. Knockout of both DCN and BGN in mice was associated with a heightened risk of dystocia and delayed labor onset, which may be underreported in EDS women due to their widespread prevalence and easy treatment [91]. While uterine tissue failure was dependent primarily on DCN, a fall in spontaneous uterine contractile amplitude and oxytocin-induced contractile force was noted in the absence of either SLRP [91]. TGFβ was downregulated in the BGN-null uterus, suggesting its role in BGN-mediated regulation of uterine muscle. There was insufficient evidence for compensation between DCN and BGN on a transcriptional or protein level in the uterus. Overall, the *Bgn^−/−^/Dcn^−/−^* mouse presents a good model for testing treatments for human dystocia [91].

Among female mice carrying wild-type and null alleles of *DCN/BGN*, the risk of PTB was inversely related to the number of wild-type *DCN* and *BGN* alleles per genotype. Decreased expression of both DCN and BGN was associated with increased risk of PTB, which was protected against by the presence of at least two wild-type SLRP alleles. Fewer offspring with the *Bgn^−/−^/Dcn^−/−^* genotype than anticipated were found after birth [92]. While mouse placental DCN and BGN protein levels were upregulated in each other’s absence, compensation likely occurs after post-transcriptional processes in the placenta. While the same was not reported in the mouse fetal membranes [92], a later study uncovered transcriptional compensation only in the absence of inflammation. This loss of compensation due to the absence of these SLRPs shortened the time between infection and PTB [133]. Consistent with other studies [91], the differential impact of these SLRPs on MMP-8 and collagen α1VI levels during inflammation [133] and differences in developmental regulation [92] suggest that the two SLRPs work through distinct pathways.

## 10. Conclusions

Decidua-derived DCN plays important paracrine roles in trophoblast stem-cell renewal, differentiation, migration, and invasiveness in normal pregnancy, and its overproduction is associated with PE. The inhibitory roles of DCN in vascular endothelial cells explain the angiostatic functions of DCN in PE. The roles of DCN in maturation of fetal membranes by virtue of promoting collagen fibrillogenesis explain the association of *DCN* mutations with PPROM, a feature of EDS. BGN appears to play a minor compensatory role in some of these pathologies such as PPROM.

### Unanswered Questions

(1) More research in needed to examine whether an elevation of plasma DCN is a predictive biomarker of PE and a subset of FGR in ethnic groups other than Caucasian (such as African American, North American indigenous, Indo-Aryan, and Indo-Dravidian) populations. (2) DCN fragments (peptides belonging to LLR5 domain) were shown to be anti-angiogenic [129] and migration-inhibitory for EVT cells [44]. Whether these peptides are predictive biomarkers for preeclampsia remains to be investigated. Biological functions of matricryptic fragments from other SLRPs in the placenta remain an open area for future studies. (3) While DCN has been shown to act as an inhibitory ligand by binding to several TKRs such as EGFR, IGFR-1, VEGFR-2, and MET, the possibility of DCN action by binding to other TKRs (such as platelet-derived growth factor receptor (PDGFR), HER2, ROS1, ALK, and RET) needs to be examined. (4) Possible functions of BGN in human or murine decidual cells need be explored by knocking out *BGN* in ESCs. (5) The functional significance of DCN expression in hyper-invasive placental trophoblast remains to be explored. (6) The molecular mechanisms of resistance of choriocarcinoma cells to invasion-restraining functions of DCN need to be examined.

## Figures and Tables

**Table 1:** A comprehensive classification of SLRPs.

Class	Gene Symbol	Eponym	Predominant GAG	Cys-Rich Cluster Consensus	Chromosome Location
I	DCN	Decorin	DS *	C_X3_C_X_C_X6_C	12q21.33
	BGN	Biglycan	CS ^#^		Xq28
	ASPN	Asporin			9q22.31
	ECM2	Extracellular matrix protein 2			9q22.31
	ECMX	ECM2-like protein, X chromosome			Xq28
II	FMOD	Fibromodulin	KS **	C_X3_C_X_C_X9_C	1q32.1
	LUM	Lumican	KS **		12q21.33
	PRELP	PRELP			1q32.1
	KERA	Keratocan	KS **		12q21.33
	OMD	Osteomodulin	KS **		9q22.31
III	EPYC	Epiphycan	DS */CS ^#^	C_X2_C_X_C_X6_C	12q21.33
	OPTC	Opticin			1q32.1
	OGN	Osteoglycin			9q22.31
IV	CHAD	Chondroadherin		C_X3_C_X_C_X6–17_C	17q21.33
	NYX	Nyctalopin			Xp11.4
	TSKU	Tsukushi			11q13.5
V	PODN	Podocan		C_X3–4_C_X_C_X9_C	1p32.3
	PODNL1	Podocan like 1			19p13.12

* Dermatan sulfate; # chondroitin sulfate; ** keratan sulfate. SLRPs are divided into five classes. Class I to III are canonical SLRPs and IV and V are non-canonical. Table was modified from [11,15]. The consensus for the N-terminal Cys-rich cluster is shown. where X represents any amino acid, while C represents a cysteine amino acid. Predominant GAG and chromosomal location of the various SLRP genes is also shown.

**Table 2:** Distribution and functions of DCN and BGN in the pregnant uterus and placenta.

SLRP	Distribution	Species	Function	Mode of Action
DCN	Decidua	Human	Restraining trophoblast invasion, migration, invasiveness, and endovascular differentiation	EGFR, IGFR-1, and VEGF-2
			Decidual cell maturation	HAND2 and PGR
		Mice	Collagen fibrillogenesis	Collagen
	Decidua/placenta (fetal mesenchyme)	Human	Restraining trophoblast stem cell self-renewal and differentiation	N/A *
	Placenta (fetal mesenchyme)	Cow	Restraining proliferation, migration, and angiogenesis	
		Rat	Inducing flexibility	Collagen
	Fetal membranes	Human	Matrix remodeling and structural stability	N/A
		Mice		TGFβ, Smad, MMPs, and TIMPs
	Cervix	Human	Collagen organization	Collagen
		Rat		
	Myometrium	Ewe	Myometrial activation	N/A *
	Endometrium	Human	Matrix remodeling during the menstrual cycle	
BGN	Decidua	Mice	Collagen fibrillogenesis	Collagen
	Fetal membranes	Human	Matrix remodeling and structural stability	N/A *
		Mice		TGFβ, Smad, MMPs, and TIMPs
	Cervix	Human	Collagen organization	Collagen
	Myometrium	Mouse	N/A *	N/A *
	Endometrium	Human	Matrix and tissue organization during the menstrual cycle	
		Rhesus monkey		
DCN/BGN	Fetal membranes/placenta	Mice	Achievement of term gestation, with compensatory upregulation	
	Uterus		Uterine function during parturition, with partial compensation of DCN by BGN	TGFβ

* N/A: not known at present.

## Abbreviations

α-hCG, human chorionic gonadotrophin alpha chain; BGN, biglycan; BMI, body mass index; BMP, bone morphogenetic protein; CCL, C–C motif chemokine ligand; CS, chondroitin sulfate; CXCL, C–X–C motif chemokine ligand; DCN, decorin; DS, dermatan sulfate; E2, estradiol; ECM, extracellular matrix; EDS, Ehlers–Danlos syndrome; EGFR, epidermal growth factor receptor; EMT, epithelial–mesenchymal transition; EOS, early-onset; ERK, extracellular signal-regulated kinase; EVT, extravillous trophoblast; FGR, fetal growth restriction; FN, fibronectin; GAG, glycosaminoglycan; GD, gestational day; HAND2, heart and neural crest derivatives-expressed protein 2; HC II, heparin cofactor II; HESC, human endometrial stromal cell; h-hCG, hyperglycosylated human chorionic gonadotrophin; HIF-1α, hypoxia-inducible factor 1 alpha; HMVEC, human microvascular endothelial cell; hPL, human placental lactogen; IGFBP-1, insulin-like growth factor-binding protein 1; IGFR, insulin-like growth factor receptor; IL, interleukin; LOS, late-onset; LRR, leucine-rich repeat; MAPK, mitogen-activated protein kinase; MIP-2, macrophage inflammatory protein 2; MMP, matrix metalloproteinase; MPA, medroxyprogesterone acetate; NF-κB, nuclear factor kappa B; NOX, NADPH oxidase; PBGC, placental bed giant cell; PCOS, polycystic ovary syndrome; PDGFR, platelet-derived growth factor receptor; PE, preeclampsia; PG, proteoglycan; PGR, progesterone receptor; PLEC, placental microvascular endothelial cell; PPROM, preterm premature rupture of fetal membranes; PRELP, proline/arginine-rich end leucine-rich repeat protein; PRL, prolactin; PRLR, prolactin receptor; PTB, preterm birth; SGA, small for gestational age; SLRP, small leucine-rich proteoglycans; STB, syncytiotrophoblast; TGF, transforming growth factor; TIMP, tissue inhibitors of metalloproteinase; TKR, tyrosine kinase receptor; TLR, Toll like receptor; TNFα, tumor necrosis factor alpha; TSP-1, thromobospondin-1; UA, umbilical artery; uNK, uterine natural killer cell; UV, umbilical vein; VEGFR, vascular endothelial growth factor receptor; WISP, Wnt-1-induced secreted protein.
